# Cell surface thiol-disulfide regulation in cancer: Mechanisms, implications, and theranostic strategies

**DOI:** 10.1016/j.redox.2026.104142

**Published:** 2026-03-25

**Authors:** Jost Lühle, Peter H. Seeberger, Oren Moscovitz

**Affiliations:** a Department of Biomolecular Systems, Max Planck Institute of Colloids and Interfaces, Potsdam, Germany; b Institute of Chemistry and Biochemistry, Freie Universität Berlin, Berlin, Germany; c Scojen Institute for Synthetic Biology, Dina Recanati School of Medicine, Reichman University, Herzliya, Israel

## Abstract

Redox homeostasis is frequently disrupted in cancer and contributes to tumor progression, metastasis, and therapy resistance. This review focuses on how thioredoxin-1 (TXN1), thioredoxin reductase-1 (TXNRD1), and members of the protein disulfide isomerase (PDI) family regulate thiol-disulfide balance at the cancer cell surface and how these alterations can be exploited for theranostic applications. Cancer cells typically exhibit elevated reactive oxygen species (ROS) levels that are counterbalanced by upregulation of antioxidant systems, including TXN1/TXNRD1 and PDIs, which also act at the cell surface. This activity remodels surface redox states, generating reduced microenvironments that promote invasion, metastasis, and resistance to therapy.

We summarize evidence from multiple malignancies, including breast, colon, lung, prostate cancer, and B-cell chronic lymphocytic leukemia, showing that altered exofacial thiol-disulfide states driven by TXN1 and PDI overexpression represent reproducible biochemical features of cancer progression. Building on this redox phenotype, we discuss thiol-mediated targeting strategies that enable selective delivery of small molecules, peptides, antibodies, liposomes, and nanoparticles to cancer cells. Emphasis is placed on emerging redox-responsive approaches such as cyclic oligochalcogenides, cell-penetrating polydisulfides, and redox-sensitive antibodies.

Overall, this review highlights extracellular redox regulation as a tumor-associated feature that can serve both as a biomarker and as a basis for next-generation cancer theranostics, offering complementary opportunities beyond antigen-specific strategies.

## 1 Introduction

### 1.1 Redox homeostasis in cancer

**Fig. 1:**
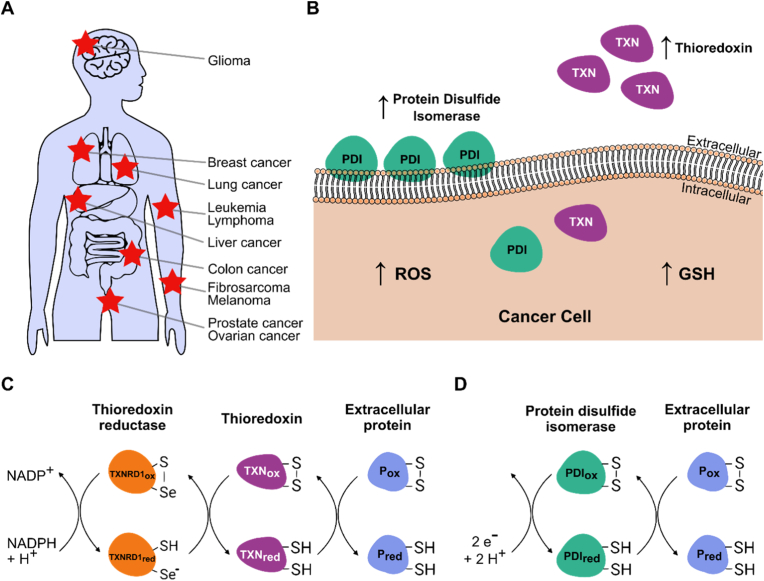
**Cancer redox biology. A**, Cancer types that were described to overexpress oxidoreductases from the thioredoxin system (TXN1) or the protein disulfide isomerase family (PDI) are indicated by red stars at their respective primary tumor site in the human body. **B**, A schematic cancer cell is shown, emphasizing the increased secretion and cell surface retention of TXN1 and PDI, as well as the intracellular accumulation of reactive oxygen species (ROS) and reduced glutathione (GSH). **C-D**, The mechanism of extracellular protein disulfide reduction by the thioredoxin system (**C**) and PDIs (**D**) is shown (“2 e^−^ + 2 H^+^” represents an unspecified extracellular reducing input to surface PDI). Abbreviations: NADP – oxidized nicotinamide adenine dinucleotide phosphate, NADPH – reduced nicotinamide adenine dinucleotide phosphate, P – protein, TXNRD1 - thioredoxin reductase-1, ox – oxidized, red – reduced.

Cellular redox potential plays a vital role in both healthy and malignant tissues. Governed by tightly regulated electron flux, it influences various biological processes, including cell-cell communication and signaling [1,2]. The net redox potential of a cell, which can be determined experimentally, is an integration of the redox states of individual proteins and organelles and the abundance of pro- and antioxidant species [3,4]. These individual redox states, whether intra- or extracellular, can vary significantly in a highly localized and specific manner [1,5,6]. In cancer, malignant transformation often disrupts cellular redox homeostasis, leading to dramatic changes in net cellular redox potential and subcellular redox states (Fig. 1A–B) [5,6]. These alterations can simultaneously promote and result from tumorigenesis and provide valuable insights into cancer stage and progression [5,7].

Elevated production of reactive oxygen species (ROS) is a hallmark of cancer, leading to oxidative stress [7]. Superoxide radicals (O_2_∙^-^) and hydrogen peroxide (H_2_O_2_) are the most abundant intracellular ROS and are byproducts of the hypermetabolism of cancer cells [7]. ROS play a dual role in cancer biology: they are pro-tumorigenic and increase proliferation and survival but can also induce apoptosis and increase cell vulnerability when their levels become uncontrollable [5,7]. Consequently, cancer cells must tightly regulate their redox environment to survive. Many cancers overexpress antioxidant systems to mitigate oxidative stress, both enzymatic and non-enzymatic. The primary non-enzymatic intracellular antioxidant is reduced glutathione (GSH), whose concentration is elevated in cancer cells [8]. Other non-enzymatic representatives include low-molecular-weight thiols, such as the cysteine/cystine system, and some vitamins [[9], [10], [11]]. Enzymatic antioxidants play an important role in maintaining redox homeostasis. Superoxide dismutase (SOD) and catalase (Cat) are two major ROS-scavenging enzymes. SOD catalyzes the dismutation of superoxide radicals to oxygen and hydrogen peroxide, whereas Cat dismutates hydrogen peroxide to oxygen and water [10]. Other enzymatic factors that can reduce hydrogen peroxide are peroxiredoxins and glutathione peroxidases [5,9].

Building on the tight intracellular regulation of redox homeostasis, cancer cells also influence the extracellular redox landscape to support their survival and progression. The mechanisms that sustain redox signaling involve secreted factors that shape the tumor microenvironment (TME). Extracellular vesicles (EVs), including exosomes and microvesicles, carry diverse bioactive cargo such as proteins, RNA, lipids, and metabolites, which modulate recipient cell behavior and mediate intercellular communication in cancer [12,13]. Some EVs contain redox-active or ROS-generating enzymes, such as members of the NADPH oxidase (NOX) family, suggesting that EVs can directly contribute to extracellular reactive oxygen species (ROS) generation and redox signaling [13]. Through this cargo transfer, EVs can influence metabolism, immune modulation, and redox homeostasis in the TME, promoting tumor proliferation, metabolic reprogramming, immunosuppression, and therapy resistance [14]. Damage-associated molecular patterns (DAMPs) released by stressed or dying cells also influence extracellular redox states. Several DAMPs, including HMGB1, are redox-sensitive: their release, structural conformation, and activity depend on their oxidative state, and extracellular oxidation can alter their immunogenicity and downstream signaling [15,16]. Oxidized or disulfide-containing DAMPs in the extracellular milieu can alter local redox status, influence immune cells and inflammation, and thereby contribute to a redox-relay network in the TME [16]. While extracellular vesicles, DAMPs, and other oxidized secreted factors collectively shape the redox landscape of the tumor microenvironment, specific enzymatic systems also play a critical role in modulating extracellular redox states. Among these, the oxidoreductases of the thioredoxin superfamily stand out as key regulators of protein thiol-disulfide exchange, influencing not only intracellular processes but also cell surface redox states.

These enzymes, which are characterized by a pair of reactive cysteine residues in their active centers, catalyze the reduction, oxidation, and isomerization of protein cysteines [1,17]. Their role in protein folding is situated primarily in the cytosol or endoplasmic reticulum (ER), where they can act as chaperones [[18], [19], [20]]. Interestingly, some thioredoxin superfamily members are exported to the cell surface, where they function as extracellular thiol-modifying enzymes, thereby influencing critical biological processes [1,20].

### 1.2 The thioredoxin system

The thioredoxin system is a key cellular antioxidant and redox regulatory system, comprising thioredoxin (TXN) and thioredoxin reductase (TXNRD) [1,21]. It plays a critical role in maintaining cellular redox homeostasis by reducing disulfide bonds in target proteins to their free-thiol forms. This mechanism involves TXN reduction by TXNRD in an NADPH-dependent reaction. Reduced TXN, in turn, donates electrons to disulfide-containing substrates, thereby regenerating their reduced forms (Fig. 1C) [22]. This system is crucial for DNA synthesis, repair, and the regulation of redox-sensitive signaling pathways [23]. Thioredoxin is present in two isoforms in humans that display distinct localizations, with TXN1 and TXNRD1 acting predominantly in the cytosol, whereas TXN2 and TXNRD2 are confined to the mitochondria [24]. Beyond its intracellular role, TXN1 is secreted to the cell surface under certain conditions through a non-classical, leaderless pathway and independently of the ER-Golgi route [[25], [26], [27]]. Its release is enhanced by oxidative and inflammatory stimuli and can involve caspase-1-dependent regulation, indicating an active stress-responsive export mechanism [25]. In contrast, secretion of thioredoxin reductase (TXNRD1) follows a classical Golgi-dependent pathway [28]. On the cell surface, TXN1 exhibits various functions, including acting as a cytokine-like molecule that modulates immune responses, protecting cells from extracellular oxidative damage, and influencing cell signaling [29,30]. This dual intracellular and extracellular functionality underscores its importance in maintaining the redox balance and modulating physiological and pathological processes.

Elevated levels of TXN1 and TXNRD1 have been observed in several types of cancer and are associated with tumor growth, resistance to apoptosis, and enhanced metastatic potential [22,[31], [32], [33], [34]]. Thus, the thioredoxin system is being explored as a therapeutic target [35,36].

### 1.3 The protein disulfide isomerase family

The protein disulfide isomerase (PDI) family comprises a group of thiol-disulfide oxidoreductases primarily located in the endoplasmic reticulum (ER), where they facilitate the formation, isomerization, and reduction of disulfide bonds in nascent proteins [18,37]. The PDI family comprises more than 20 members, including PDIA2-6 [38], which will be discussed later in the context of cancer. The activity of PDIs involves two conserved thioredoxin-like domains, which contain active-site cysteine residues. These cysteines can alternate between the reduced and oxidized states, enabling PDIs to catalyze the rearrangement of incorrect disulfide bonds and assist in oxidative protein folding [37]. In addition to their catalytic role, PDIs also act as molecular chaperones, preventing protein aggregation during folding [[39], [40], [41]]. However, PDIs also play a significant role beyond the ER. They are found on the cell surface, where they participate in processes such as integrin-mediated adhesion and cell signaling [42]. Cell surface PDIs contribute to the reduction of extracellular disulfide bonds (Fig. 1D), modulating receptor function, and facilitating processes such as platelet aggregation, immune responses, and viral entry into host cells [[43], [44], [45], [46]].

The localization of PDI to the cell surface, despite its classical retention in the endoplasmic reticulum via the C-terminal KDEL motif, can occur through multiple mechanisms. Recent work shows that PDIs such as PDIA1, PDIA3, and PDIA6 associate with the plasma membrane through a KDEL endoplasmic reticulum protein retention receptor 1 (KDELR1)-dependent trafficking route, where KDELR1 itself dynamically translocates to the cell surface and can deliver PDI directly to membrane-proximal substrates. Notably, the KDEL sequence is required for cell-surface association but prevents secretion, suggesting that transport occurs via receptor-mediated surface delivery rather than uncontrolled escape from ER retention [47]. An earlier study also demonstrates that KDEL-bearing proteins including PDI can indeed appear on the extracellular surface of mammalian cells, where newly synthesized pools preferentially reach the membrane via a brefeldin-A-sensitive pathway, supporting involvement of the canonical ER-to-Golgi secretory route [48]. Once at the plasma membrane, PDI forms complexes with surface proteins, enabling redox-mediated regulation of membrane receptors and adhesion processes rather than acting as a freely secreted enzyme [48]. Together, these findings clarify that PDI surface localization is an active, receptor-assisted trafficking process coupled to specific membrane interactions, rather than passive leakage from the ER.

The PDI family has been implicated in tumor progression and survival. PDIs support the high protein-folding demand of rapidly proliferating cancer cells and help manage ER stress that arises from their dysregulated metabolism [18,49]. Elevated expression of certain PDIs (namely PDIA1, -3, -4, and -6) has been observed in various cancers and is associated with poor prognosis as it enhances tumor growth, invasion, and resistance to apoptosis [50,51].

To cope with oxidative stress conditions, many cancers upregulate extracellular oxidoreductases and other redox-active factors that alter their cell surface redox states [18,22]. While the underlying mechanisms remain incompletely understood, accumulating evidence links these surface redox alterations with cancer phenotype, progression, and stage [22,49]. Although enzymes such as TXN1 and PDIs have been individually reviewed as drug targets, no existing article synthesizes how their dysregulated expression remodels cancer cell surface redox states and constitutes an independent theranostic opportunity [18,52]. Moreover, despite growing interest in redox-reactive delivery platforms, an integrative analysis of their shared mechanisms, biomedical advantages, and translational limitations is still missing. Thus, this review aims to provide a comparative perspective on how altered extracellular redox states can be leveraged for tumor-specific therapy and diagnosis. To achieve this, we first review the literature on the upregulation and biological roles of extracellular redox-active enzymes across malignancies and how they reshape the extracellular redox milieu. In the second part, we outline the mechanistic principles by which these alterations can be exploited for redox-guided theranostics, followed by a comparative overview of current redox-reactive strategies and technological platforms.

## 2 Cell surface redox biology in cancer

### 2.1 Thioredoxin remodels cellular redox states and drives cancer progression

In various cancers, the thioredoxin system plays a crucial role in regulating redox states, influencing key processes, such as invasion, migration, and resistance to therapies. In a model for triple-negative breast cancer, the increased expression of TXN1 and TXNRD1 correlates with enhanced cell invasion and migration [33]. Interestingly, this phenotype can also be induced by exogenously added TXN1 and a redox-inactive TXN1 mutant has no effect on breast cancer invasion and migration. Furthermore, high TXN1 and TXNRD1 levels are associated with poor overall survival, disease-free survival, and distant metastasis-free survival in primary breast cancer patients [33]. On the other hand, high TXN1 expression was also associated with resistance to chemotherapy, particularly to docetaxel, making this enzyme a potential clinical marker for predicting responsiveness to docetaxel treatment in primary breast cancer patients [34].

In human colon epithelial cancer, proliferative cells show the most reduced cellular redox state, whereas an increase in TXN1 expression can be observed as cells progress from proliferation to differentiation, suggesting that TXN1 may play a role in maintaining redox-sensitive cellular functions and supporting differentiation under increasingly oxidative conditions [53].

Similarly, non-small cell lung carcinomas (NSCLC) and prostate cancer show altered redox states, where a reduced extracellular microenvironment promotes tumor aggressiveness [31,32]. Specifically, reduced redox states in lung cancer correlate with increased cancer aggressiveness, presumably driven by the overexpression of extracellular TXN1 and non-protein thiols (e.g., GSH), compared to non-neoplastic tissues [32]. In prostate cancer, upregulation of extracellular TXN1 compared to that in healthy prostate cells and a more reduced extracellular redox state are linked to enhanced invasion and migration [31]. Elevated extracellular GSH/GSSG ratios and superoxide levels further promote this invasive behavior by remodeling the cell surface and extracellular redox states [54]. Similarly, aggressive and metastatic prostate cancer cells exhibit a more reduced state than less aggressive cells, a condition that is influenced by increased levels of GSH, glutathione-related proteins, and antioxidant defenses, and contributes to cancer cell survival and resistance to oxidative stress [55].

Current knowledge indicates that upregulation of the thioredoxin system contributes to the formation of a more reduced cell surface microenvironment, which is correlated with tumor progression, invasion, and aggressiveness (Table 1) [15,31,32,53]. Overall, the redox balance at the cancer cell surface, influenced by thioredoxin, plays a major role in cancer progression and response to therapy [33,34], making it a potential target for therapeutic intervention.

**Table 1:** Cell surface redox states by cancer, cancer phenotype, and associated redox-active enzymes.

Cancer	Cancer Phenotype	Net Cell Surface Redox State	Associated Enzymes and non-enzymatic Antioxidants	References
**B Cell Chronic Lymphocytic Leukemia (B-CLL)**	n.a.	Reduced Increased exofacial thiols	Protein Disulfide Isomerase (PDI)	Täger et al. [56] Söderberg et al. [57]
**Breast Cancer**	Metastatic	Reduced Increased exofacial thiols	Thioredoxin (TXN1) PDI	Bhatia et al. [33] Stojak et al. [58] Popielarski et al. [59] Lee et al. [60]
**Breast Cancer**	Docetaxel-resistant	n.a.	TXN1	Kim et al. [34]
**Colon Cancer**	Proliferating	Reduced	TXN1 Glutathione (GSH)	Nkabyo et al. [53]
**Fibrosarcoma**	n.a.	Reduced Increased exofacial thiols	PDI	Jiang et al. [61]
**Glioma**	Aggressive Enhanced migration and invasion Poor prognosis	n.a.	PDI	Goplen et al. [62] Zou et al. [63]
**Lung Cancer**	Advanced Aggressive	Reduced	TXN1 GSH	Ceccarelli et al. [32]
**Lung Cancer**	Cisplatin-resistant	n.a.	PDI	Tufo et al. [64]
**Lymphoma**	n.a.	Reduced Increased exofacial thiols	n.a.	Goerdeler et al. [65]
**Lymphoma**	n.a.	Oxidized	Gamma-Glutamyl Transpeptidase (GGT)	Dominici et al. [66]
**Melanoma**	Aggressive	Oxidized	GGT	Maellaro et al. [67] Paolicchi et al. [68]
**Prostate cancer**	Aggressive Enhanced migration and invasion	Reduced	TXN1 GSH	Chaiswing et al. (2007) [55] Chaiswing et al. (2008) [54] Chaiswing et al. (2012) [31]

### 2.2 Protein disulfide isomerases reduce the cancer cell surface and promote metastasis

While the role of intracellular PDI has been studied more extensively, cell surface-associated forms remain poorly investigated. However, increasing evidence points towards PDI as a central player in modulating extracellular redox states. Cell surface PDI retention in T cells, for example, was shown to increase the number of exofacial thiol groups [45]. Furthermore, the involvement of PDI in integrin activation depends on thiol group exposure after activation, as observed in human fibroblasts and HEK cells [42,69]. PDI family members are upregulated in a variety of cancer types, with implications in tumor biology. Kuo et al. [70] show that PDIA4 is upregulated in several standard human cancer cell lines, including breast, ovarian, and liver cancers, as well as leukemia, and is increased in the tissues of lung adenocarcinoma patients. Furthermore, PDIA4 promotes cancer proliferation in Jurkat cells (T cell leukemia) by negatively regulating the procaspase pathway [70].

In B cell chronic lymphocytic leukemia (B-CLL), malignant cells express high levels of membrane-associated PDI that closely correlate with elevated surface thiols, a feature that distinguishes them from healthy B cells. This aberrant thiol elevation suggests that PDI plays a role in regulating the redox state of exofacial membrane proteins and consequently influences drug sensitivity. Notably, PDI inhibition appears to further increase membrane thiol levels, indicating a complex regulatory mechanism that could impact therapeutic responses. These insights highlight PDI as a potential target for modulating treatment sensitivity in B-CLL [56]. Another study on B-CLL described a physical and functional association between PDI, TXN1, and tumor necrosis factor receptors (TNFRs) on the surface of CLL cells, which enhances the TNF-mediated signaling pathways that promote leukemia cell survival [57]. These factors are significantly overexpressed in CLL cells compared to healthy B cells, suggesting a critical role in the pathophysiology of the disease. Notably, inhibition of PDI using antibodies or specific inhibitors reduces TNF release and decreases leukemia cell viability. Additionally, the tumor microenvironment contributes redox-active components, such as cysteine and TXN1, which protect leukemic cells from apoptosis and further promote their survival, particularly in aggressive IGHV-unmutated CLL cases [57].

In human breast adenocarcinoma cell lines, extracellular PDI isoforms are central to the processes that underlie tumor metastasis. PDIA1, for example, modulates cancer cell adhesion and transmigration by regulating cell surface thiols through disulfide exchange, a process critical for integrin activation. Inhibition of PDI markedly reduced cell adhesion to key extracellular matrix components, such as collagen and fibronectin [58]. Moreover, thiol-disulfide exchanges facilitate the activation of integrin β-chains, which in turn drive cancer cell adhesion, migration, and metastasis. Notably, metastatic breast cancer cell lines showed significantly higher levels of exofacial thiols than non-metastatic lines. Blocking these thiols or inhibiting PDI effectively diminishes these malignant behaviors [59]. In tissues from invasive ductal carcinoma patients, the progressive overexpression of PDIs, especially PDIA3, from early to advanced metastatic stages underscores their value as prognostic markers and therapeutic targets [60].

In human fibrosarcoma cells, the modulation of PDI expression significantly affects the redox balance of the cell surface. Cells overexpressing PDI exhibited a 41–50% increase in exofacial thiols, whereas those with reduced PDI levels showed a 29–33% decrease. This correlation between PDI expression and the abundance of exofacial protein thiols underscores the critical role of the enzyme in maintaining redox homeostasis [61].

In glioma, PDIs are upregulated in cells that exhibit invasive and migratory properties [62]. Furthermore, diffuse glioma patients with high PDI expression show a poor prognosis [63]. The use of PDI inhibitors, including bacitracin and monoclonal antibodies, significantly reduced the migration of glioma cells [62].

In lung adenocarcinoma, cells that develop resistance to cisplatin-induced apoptosis exhibit overexpression of PDIA4 and PDIA6. By blocking distinct pre-mitochondrial pathways, PDI isoforms foster an anti-apoptotic environment that protects cancer cells from chemotherapy-induced cell death. Therefore, targeting PDIA4 and PDIA6 may offer a viable strategy for overcoming drug resistance in lung cancer [64].

### 2.3 Gamma-glutamyl transpeptidase acts as cell surface Prooxidant

The literature presented so far indicates that cancers, in general, but especially aggressive and metastatic phenotypes, often exhibit reduced cell surface redox states; however, in cancers with gamma-glutamyl transpeptidase (GGT) upregulation, these states are shifted towards a more oxidized condition. GGT plays a key role in glutathione metabolism by transferring the gamma-glutamyl group to other amino acids. This process generates hydrogen peroxide (H_2_O_2_) as a by-product, which can increase oxidative stress [71]. GGT is overexpressed in several cancers, including lung, colon, and leukemia [[72], [73], [74], [75], [76], [77]].

In a histiocytic non-Hodgkin lymphoma cell line, GGT activity leads to a decrease in exofacial thiols, suggesting that GGT can promote a more oxidized surface redox state [66]. In contrast, a later study found that B cell non-Hodgkin lymphoma cell surfaces expose higher exofacial thiol levels [65], indicating that the individual redox potential of different non-Hodgkin lymphoma subtypes might be highly dynamic and depends on the specific type and stage.

GGT activity is also correlated with aggressive tumor behavior in melanoma. Increased GGT activity generates H_2_O_2_, which in turn reduces the levels of exofacial protein thiols, a phenomenon potentially shared by GGT-positive tumors [67,68]. However, this effect varies among different cancers, as demonstrated by the presence of different GGT expression levels and downstream effects in clones derived from the same cell line, indicating that the role of GGT may not be uniform across all tumor types [68].

### 2.4 Conclusions

Overall, the literature demonstrates that diverse cancer types, including breast cancer, B-CLL, glioma, colon, lung adenocarcinoma, melanoma, prostate cancer, and fibrosarcoma, share a common feature: their cell surface redox states are actively remodeled by extracellular oxidoreductases, most prominently thioredoxin (TXN1) and protein disulfide isomerases (PDIs) (Fig. 1). Despite differences in tumor origin, these enzymes converge on a similar outcome: the generation of aberrant exofacial thiol levels and shifts in extracellular redox potential, which in turn promote phenotypes with enhanced adhesion, invasion, migration, and therapy resistance [[31], [32], [33],[53], [54], [55], [56], [57], [58], [59], [60], [61], [62], [63], [64], [65]]. Importantly, this redox remodeling is not unique to malignant cells; healthy activated lymphocytes and platelets also transiently expose elevated surface thiols during states of high motility and activation [[78], [79], [80], [81]], underscoring that altered redox states reflect a functional phenotype rather than a cell type-specific property. Cancer appears to exploit this physiological mechanism, thereby supporting persistent tissue invasion and the formation of metastases.

Across tumor types, reduced surface redox states emerge as a consistent feature of aggressive disease, increased metastatic potential, and poor patient prognosis. Conversely, gamma-glutamyl transpeptidase (GGT)-positive tumors (e.g. subsets of melanoma, non-Hodgkin lymphoma, or leukemia) illustrate that some cancers adopt more oxidizing surface conditions due to GGT activity [[66], [67], [68]]. These discrepancies highlight that cancer-associated redox remodeling is context-dependent and shaped by tumor genetics, microenvironment, and differentiation states.

A critical evaluation of the existing data highlights several important limitations. First, most evidence arises from cell line-based studies, where redox states may differ substantially from those in primary tumors. Second, *in vivo* validation is limited, and the tumor microenvironment, which can provide a wide range of redox-active molecules, likely exerts strong influence on extracellular redox states but is rarely accounted for in the presented literature. Third, mechanistic links are often incomplete, because many studies quantify either enzyme expression (e.g. PDI or TXN1 levels) or exofacial thiols or redox potentials, but not in an integrated manner, making causality difficult to conclude. Despite these constraints, a general trend emerges in which reduced cell surface microenvironments are correlated with aggressive and invasive cancer phenotypes, as well as increased metastasis (Table 1).

Thus, targeting enzymes and mechanisms that regulate redox states, such as TXN1 and PDI, represents a promising strategy for cancer therapy and could potentially improve treatment outcomes by modulating tumor-specific redox conditions. Furthermore, altered redox states represent promising therapeutic targets in cancer, offering a biochemical signature rather than a specific antigen that may help distinguish tumor cells from healthy tissues. Targeting such a broad, tumor-associated property might offer advantages over conventional therapies that rely on distinct antigens, which can vary between cancer types and patients or can be suppressed and downregulated to evade the immune system [[82], [83], [84]]. Although numerous reviews have explored the manipulation of tumor-associated oxidoreductases by small-molecule inhibitors for cancer therapy [18,22,35,50,51], this review focuses on a distinct approach: harnessing the altered extracellular redox milieu as a therapeutic and diagnostic target. In the following section, we will focus on such strategies that target cancer redox states directly using chemical and biological approaches.

## 3 Redox-targeted cancer theranostics

### 3.1 Thiol-mediated uptake

When targeting cancer cells through their altered extracellular redox milieu, a cellular uptake mechanism, denoted by thiol-mediated uptake, is of major interest. It relies on the reactivity of the thiol groups to facilitate molecular transport across biological membranes. At its core, this mechanism exploits thiol-disulfide exchange reactions [85], leveraging the reversible nature of disulfide bonds to mediate the entry of a wide range of biomolecules, including peptides, antibodies, proteins, drugs, and nanoparticles [65,86]. This process is driven by the intracellular redox environment, where high concentrations of reducing agents, such as glutathione, promote disulfide bond cleavage and molecular release [87]. This dual functionality of cellular uptake and intracellular release makes thiol-mediated strategies particularly attractive for applications in targeted drug delivery, nanomedicine, and molecular diagnostics.

**Fig. 2:**
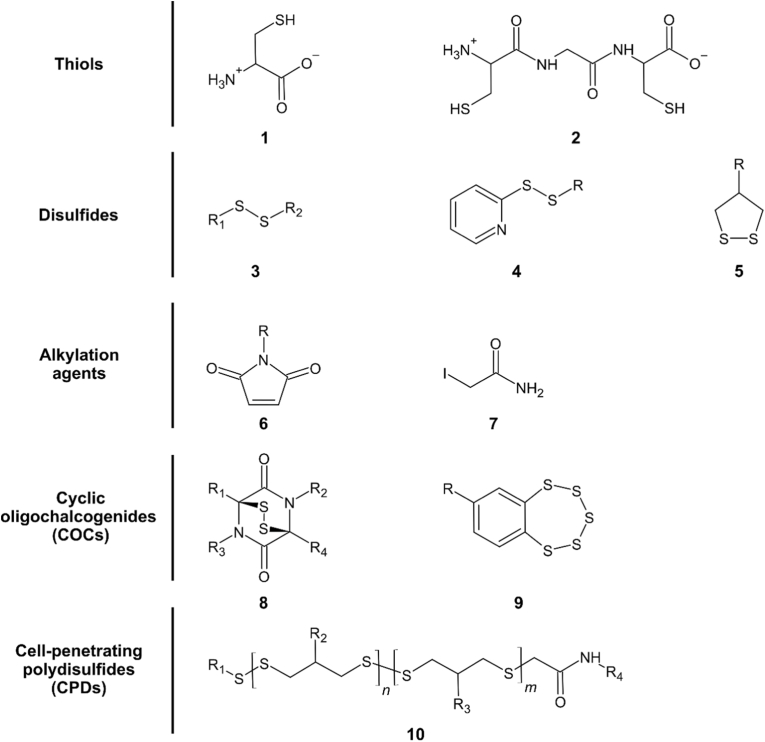
**Examples of chemical compounds used for redox-directed targeting of cancer cells and thiol-mediated uptake**. Thiols: cysteine (1), and biscysteines like the cysteine-glycine-cysteine (CGC) motif (2). Disulfides: biological disulfides (3), with R_1_ and R_2_ being a peptide or protein, pyridyl disulfide (4), and dithiolane (5). Alkylation agents: maleimide (6), and iodoacetamide (7). Cyclic oligochalcogenides (COCs): epidithiodiketopiperazines (8), and benzopolysulfanes (9). Cell-penetrating polydisulfides (CPDs): exemplary dithiolane-derived CPD (10).

Thiol-mediated uptake is a dynamic cellular entry process facilitated by compounds such as disulfides, oligochalcogenides, and their functional analogs [86,88]. This mechanism is rooted in dynamic covalent exchange, predominantly involving thiol-disulfide interactions, which can occur before or during the translocation of molecules across the cellular membranes [89]. Critical to this process is the reactivity of chalcogenides, which can trigger sulfur exchange cascades, enabling direct translocation of substrates into the cytosol or their transport through endocytic pathways [65,86,90]. Cell-penetrating polydisulfides [87,91] and cyclic oligochalcogenides [92,93] (Fig. 2) are preferred scaffolds that demonstrate efficient uptake because of their ability to react with multiple cell-surface thiols. However, it was shown that also simpler structures containing only single thiol or disulfide functional groups were efficiently taken up by cells in a thiol-dependent manner [65,94].

In the context of cancer, thiol-mediated uptake is particularly relevant because many cancers, for example B cell chronic lymphocytic leukemia [56,57], metastatic breast cancer [59], fibrosarcoma [61], and B cell lymphoma [65], display an increased abundance of exofacial protein thiols and are considered to have a higher plasticity of cell-surface redox states [6]. Together, these features suggest that thiol-reactive agents operate more efficiently in malignant cells than in their healthy counterparts. We hypothesize that elevated levels of accessible surface thiols in cancer may provide more reactive sites for initial thiol–disulfide exchange, while enhanced redox plasticity may accelerate subsequent exchange cascades, thereby facilitating overall uptake. Whether this mechanism confers cancer-specific advantages over healthy tissues remains to be determined systematically. The following sections focus on different chemical and biological platforms to exploit the altered cell surface redox states of cancer for theranostics.

### 3.2 Small molecules and inorganic compounds

Thiol- and disulfide-reactive functional groups form the core of strategies targeting extracellular redox states in cancer (Fig. 2). Classical thiol-disulfide exchange reactions can be facilitated by functionalizing compounds with cysteines, bicycles, or synthetic disulfides such as pyridyl disulfide. Notably, the targeting efficiency increases with greater ring strain in the disulfide, making cyclic oligochalcogenides (COCs) preferring scaffolds [95]. The simplest COC is dithiolane, which is derived from asparagusic acid, but uptake efficiency can be further enhanced by incorporating moieties with even smaller carbon-sulfur-sulfur-carbon (sulfur) dihedral angles [96,97]. Another class of redox-reactive molecules is cell-penetrating polydisulfides (CPDs), which can be generated via substrate-initiated polymerization of COCs [98]. Furthermore, exofacial thiols can be targeted using alkylating agents such as maleimides and iodoacetamide [90,99].

The design and improvement of COC scaffolds have made significant progress in recent years. Epidithiodiketopiperazines (ETPs, Fig. 2), for example, exhibit highly efficient thiol-mediated uptake due to their extreme ring strain, outcompeting the uptake efficiency of dithiolanes by 20-fold [97]. Furthermore, ETP-functionalized molecules are internalized by cells independent of classical endocytosis pathways, presumably because of multitarget binding induced by molecular hopping mechanisms [97]. Another class of COCs with increased ring strain is benzopolysulfanes (BPS, Fig. 2). BPS was used successfully for endocytosis-independent thiol-mediated uptake, for example, to deliver multifunctionalized streptavidin into cells. The multiple cargos of such streptavidin molecules enable the targeting of exofacial thiols, intracellular targeting, and simultaneous delivery of drugs or fluorophores, making them a versatile platform for theranostic purposes in cancer, as shown in human breast adenocarcinoma and cervical carcinoma cell lines [96]. BPS, like ETPs, exhibit an increased efficiency of thiol-mediated uptake compared to simpler structures, such as dithiolanes [96]. Nevertheless, dithiolane (Fig. 2) can also be successfully employed to deliver biomolecules into the cells. Maynard et al. developed a traceless dithiolane-based protein tag attached to cargo via esterification [100]. This conjugate facilitates the thiol-mediated uptake of a protein of interest and ensures the intracellular removal of the tag by cytosolic esterases. Thus, traceless delivery of protein cargoes is possible, ensuring that they exert their native function inside cells [100]. Tagged proteins showed enhanced intracellular distribution in cancer cells compared to conventional cargoes. Using 3D spheroid models of the cervical cancer cell line HeLa, it was further confirmed that thiol-mediated uptake enables deeper tissue penetration, indicating an advantage over conventional approaches for targeting solid tumors [100].

As stated above, the increased ring strain of COCs enables more efficient thiol-mediated uptake. Another strategy to increase COCs efficiency is oligomerization and conversion into CPDs. For example, functionalizing short peptides with several asparagusic acid moieties enables direct cytosolic delivery and increases uptake efficiency in HeLa cells, comparable to conventional cell-penetrating peptides (CPPs) [92]. Another method for oligomerizing COCs is substrate-initiated polymerization, which produces CPDs as another class of thiol-reactive molecules. CPDs undergo dynamic covalent exchange cascades at the cell surface, leading to their direct translocation into cells [86,98]. After internalization, the CPDs undergo rapid depolymerization, releasing their cargo in a traceless manner. Additionally, compared to classical CPPs, CPDs are significantly less cytotoxic, suggesting that they are safer alternatives for biomedical applications [98].

Moving away from COCs and CPDs, several small molecules and inorganic compounds have been successfully used to harness the cellular redox states of cancer cells for theranostic purposes. For example, thiol-reactive carbon dots (CDs) have been used in multichannel sensor arrays to measure biological thiols in solution and in cells [101]. Highly sensitive CDs can detect different biological thiols, such as cysteine and GSH, and measure their abundance using their unique fluorescence patterns. Multichannel sensor arrays successfully discriminate healthy and cancerous cells using their thiol fingerprints, posing them as candidates for novel point-of-care diagnostics. This was demonstrated by Gao et al. via the discrimination of human hepatocellular carcinoma, colorectal carcinoma, and lung adenocarcinoma cell lines [101].

A thiol-guided MRI probe is another example of harnessing exofacial thiols in cancer cells for diagnostic purposes [102]. By functionalizing a gadolinium-based MRI contrast agent with pyridyl disulfide (Fig. 2), the probe undergoes thiol-mediated uptake upon disulfide exchange with exofacial thiols in melanoma cells. Furthermore, *in vivo* experiments demonstrated significantly increased tumor-specific tissue retention of the disulfide-functionalized MRI agent, highlighting the potential for thiol-targeted diagnosis [102].

Redox-related mechanisms also play an important role in inorganic cancer therapeutics. The chemopreventive agent selenite (SeO_3_^2−^) has been shown to undergo extracellular thiol-mediated reduction before it can be internalized into cells and mediate its cytotoxic effect [103]. Uptake efficiency is highly dependent on the abundance of extracellular thiols, indicating that cancers with a reduced extracellular microenvironment can be targeted more efficiently. Selenite might act as a prodrug activated by the reduced cancer microenvironment, rendering pre-reduction of selenite-based agents a potential strategy to tackle multidrug resistance in human lung cancer, hepatoma, and neuroblastoma cell lines [103]. This was supported by Tobe et al., who described selenodiglutathione (GSSeSG) as a primary metabolite of selenite and the main mediator of internalization and cytotoxicity in human breast adenocarcinoma cells [104]. Compared to inorganic selenite, GSSeSG showed higher intracellular accumulation and enhanced apoptosis [104].

Arsenic trioxide (ATO) is another inorganic anticancer drug with promising clinical outcomes that often suffers from poor cellular uptake [105,106]. One strategy for improving the intracellular delivery of ATO is thiol-mediated uptake. Liang et al. demonstrated that conjugating arsenic to organic sulfides and tetraacetyl-β-d-thioglucose dramatically increased its cellular uptake in a thiol-dependent manner [107]. The newly formed molecule, denoted as AcGlcAs, is a molecular mimic of auranofin, an established inhibitor of the thioredoxin system, and is considered to undergo a similar thiol-mediated uptake mechanism. AcGlcAs preferentially accumulates in a panel of 42 cancer cell lines compared to 13 healthy cell lines, indicating that thiol-mediated uptake also contributes to specificity [107].

In conclusion, thiol-reactive small molecules, especially COCs and CPDs, enable efficient thiol-mediated uptake in cancer cells. Several inorganic cancer therapeutics, such as selenite and arsenic trioxide, require extracellular thiols or benefit from thiol-mediated uptake. These findings indicate the importance of the cell surface redox states in cancer theranostics. The introduced scaffolds form the basis for the development of more complex tools such as functionalized liposomes and nanoparticles, which will be reviewed next.

### 3.3 Liposomes and nanoparticles

Liposomes are small vesicles formed from lipid bilayers. This structure, similar to that of cell membranes, enables them to deliver their cargo into the cytosol via membrane fusion or endocytosis. Several studies have demonstrated the use of functionalized liposomes for thiol-mediated targeting of cancer cells. Qualls et al. introduced cyclic disulfide lipids (CDLs) into the liposome bilayer, thereby enhancing thiol-dependent uptake into breast cancer cells [108]. CDLs are composed of dioleoylphosphatidylethanolamine (DOPE) lipids conjugated to different dithiolane-containing moieties at the hydrophilic heads, facilitating the thiol-mediated recognition of cancer cell redox states. In addition to mediating thiol-dependent cellular uptake, CDLs also facilitate the post-functionalization of liposomes assembled through disulfide exchange, enabling the decoration of vesicles with additional cargoes. Furthermore, CDL-functionalized liposomes did not cause cytotoxicity, rendering them promising nanocarriers for targeted breast cancer-specific drug delivery [108]. Li et al. used the thiol alkylation agent maleimide to functionalize liposomes, resulting in enhanced cellular uptake by cervical and breast cancer cells through thiol-mediated uptake [90,109]. Importantly, unlike conventional liposomes, maleimide-modified versions are not significantly affected by inhibitors of classical endocytic pathways, indicating their ability to circumvent lysosomal entrapment [90]. *In vivo* studies on breast cancer xenografts in mice showed that maleimide-functionalized liposomes encapsulating the anticancer drug doxorubicin exhibited an enhanced retention time at the tumor site as well as improved tumor clearance, indicating therapeutic advantages over conventional non-functionalized liposomes [109].

Nanoparticles are submicroscopic structures on the nanometer scale with a wide range of applications, including targeted drug delivery and diagnosis. Functionalization of nanoparticles with thiol-reactive groups is a promising approach for improving both their specificity and pharmacological properties. Several examples exploit the thiol-reactive properties of pyridyl disulfide (PDS), also known as pyridine-2-thiol, for functionalization of nanoparticles. The incorporation of PDS into copper-containing PEG-polymers enables the uptake of the resulting particles by several cell lines, corresponding to breast cancer, ovarian cancer, squamous cell carcinoma, colorectal cancer, and acute promyelocytic leukemia [110]. In response to intracellular GSH, the decomposed nanoparticles release a pyridine-2-thiol/copper combination, leading to selective cytotoxicity against cancer cells [110]. Another approach uses PDS to generate thiol-reactive star polymers, which can be equipped with additional functional groups such as fluorophores. The resulting multifunctional nanoparticles can be used for the quantification of exofacial thiols in different cell types, as it was shown by Glass et al. for human blood components [111]. In healthy cells, the polymers exhibit an enhanced interaction with granulocytes, monocytes, dendritic cells, platelets, and B cells [111]. Notably, thiol-reactive star polymers exhibit a significantly higher association with acute lymphoblastic leukemia (ALL) cells in patient-derived xenografts than non-functionalized polymers. Interestingly, the uptake of star polymers by cells was shown to occur primarily through dynamin-dependent clathrin-mediated endocytosis [112]. Slezak et al. exploited the thiol-reactive properties of PDS to deliver polymeric glyco-adjuvant p(Man-TLR7-PDS) towards human breast adenocarcinoma cells [113]. In addition to PDS, this complex nanoparticle contains a mannose residue and toll-like receptor 7 (TLR7) agonist. While mannose mediates lectin-dependent internalization of the polymer by antigen-presenting cells (APCs), the TLR7 agonist induces enhanced APC activation, leading to a robust antitumor immune response [113].

In addition to PDS, several other functional groups also activate nanoparticles for thiol-mediated uptake. Thiol- and disulfide-functionalized self-assembling fluorescent organic nanoparticles (FONPs) respond to protein disulfide isomerase (PDI)-mediated thiol-disulfide redox reactions [114]. Different modifications of these FONPs enabled the differential analysis of PDI activity, allowing for the sensing of both oxidase and reductase activities separately. This system was shown to be highly PDI specific and unaffected by other enzymes and proteins, highlighting its potential as a diagnostic tool for the detection of abnormal PDI activity in cancer cell lines. However, the study by Ghosh et al. only provides evidence for the use of FONPs in murine melanoma cells and application towards human cancer models remains to be tested [114].

In another approach, Zhang et al. used folic acid-functionalized disulfide-based nanoparticles (FA-DBNPs) composed of dithiolane-containing monomers that were partially modified with folic acid. These nanoparticles combine the targeting of the cancer-associated folate receptor with thiol-mediated uptake [115]. FA-DBNPs showed enhanced transcellular transportation in HeLa cells, as well as longer tumor retention and deeper tissue penetration *in vivo*. Furthermore, FA-DBNPs exhibit notable tumor clearance in mice when delivering an antisense oligonucleotide against the apoptosis inhibitor survivin [115].

The delivery of therapeutic nucleic acids via thiol-mediated uptake was also achieved by Zhu et al., who exploited cell-penetrating, disulfide-functionalized polyguanidines. These nanoparticles can enter breast cancer cells upon interaction with exofacial thiols [116]. The cage-like structure enabled the delivery of anticancer nucleic acid plasmids encoding the apoptosis-related protein P53, and KillerRed ROS-generating light-activated protein, rendering the system promising for gene/photodynamic therapy [116].

Wen et al. also exploited the underlying mechanism to generate a novel caged live cell vaccine (CLCV) against murine melanoma that relies on thiol-disulfide exchange reactions. Thiol-activated bovine serum albumin (BSA)-based nanoparticles are anchored to the surface of melanoma cells by reacting with exofacial thiols [117]. This cage-like structure restricts cancer cell proliferation, while maintaining metabolic activity. Further functionalization of the cage with a photosensitizer and subsequent photoactivation induces immunogenic cell death, resulting in the enhanced immunogenicity of CLCV. Consequently, mice vaccinated with photoactivated CLCV develop robust CD8^+^ T cell responses, providing good protection against tumor initiation and significantly reducing tumor growth in a therapeutic setting [117].

Interestingly, simple thiol modification of nanoparticles can induce thiol-mediated uptake and efficient cellular delivery. This was demonstrated by Knoll et al., who modified nanostructured lipid carries (NLCs) with thiol groups. The modified NLCs exhibited higher cellular adhesion and internalization than unmodified structures [118]. Notably, thiolated NLCs enter cells through a broad range of mechanisms, including caveolae-dependent uptake and clathrin- and caveolae-independent mechanisms. Furthermore, functionalized NLCs exhibit enhanced permeability across colorectal adenocarcinoma monolayers, presumably due to the thiol-mediated opening of tight junctions [118]. This observation suggests that thiol functionalization may contribute to deeper penetration and more homogeneous tumor distribution, even in three-dimensional tissues.

In summary, thiol-reactive liposomes and nanoparticles have a wide range of applications, from nanosensors for oxidoreductase activity to gene therapy vectors and live-cell vaccine scaffolds. Their chemical syntheses are highly versatile because of their ability to functionalize them with a wide range of simple or complex thiol-reactive moieties. However, these modifications are also applicable to biomolecules such as peptides and antibodies. The following sections review the advantages of thiol-reactive biomolecules in cancer targeting.

### 3.4 Peptides and nucleic acids

One way to functionalize peptides for thiol-mediated uptake is to conjugate the COCs to the amino acid sequence. Several studies have investigated the suitability of dithiolane (also known as asparagusic acid) for delivering peptides to cancer cells. Asparagusic acid molecules can be chemically attached to lysine side chains or incorporated into solid-phase peptide synthesis (SPPS) [119]. The introduction of asparagusic acid significantly increases the cellular uptake of peptides [119,120]. Notably, this mechanism can also be exploited to deliver therapeutic peptides, such as pro-apoptotic BH3 domain peptides, into human cervical, breast, and colorectal cancer cell lines [120]. The uptake mechanism of COC-functionalized peptides seems to be dependent on the transferrin receptor, which upon internalization interacts with asparagusic acid through cysteines 556 and 558 [120,121]. Dithiolane-modified short β-sheet peptides attached to streptavidin, the so-called dithiolane quartets, are mainly internalized in a thiol-dependent manner via the transferrin receptor, either by transcytosis or direct translocation [121]. Similar to many of the nanoparticles reviewed above, dithiolane quartets exhibit deep-tissue penetration in HeLa cells-derived multicellular spheroids *in vitro*, indicating possible advantages over conventional CPPs [121].

In addition to the functionalization of peptides with COCs, simple cysteine modification can also enable their internalization into cells via thiol-dependent pathways. Remarkably, insertion of a single cysteine into a peptide sequence can improve uptake; however, disulfide modifications have demonstrated higher efficiencies [122]. A similar behavior was observed for peptide nucleic acids (PNAs) functionalized with a single-terminal cysteine, leading to enhanced cellular uptake via endocytic pathways [94]. Modification of peptides and proteins with cysteine offers a significant advantage over synthetic COCs, as they are biocompatible and can be easily introduced into recombinant peptides or protein biosynthesis [123]. To improve the uptake efficiency of single cysteine-modified peptides, attempts have been made to incorporate biscysteines into the sequence, allowing them to form intramolecular disulfides. Here, the cysteine-glycine-cysteine (CGC) motif appeared to be the most favorable scaffold for the efficient thiol-mediated uptake of biscysteine-modified peptides [123].

### 3.5 Antibodies

Conventional antibodies can be delivered to cells via functionalization with thiol-reactive compounds. Kong et al. showed that conjugating antibodies with CPDs enabled their delivery into the cytosol of cervical and colon cancer cells via thiol-mediated uptake. After internalization, the antibody was released via GSH-dependent cleavage of the conjugate [124]. This indirect approach allows targeted delivery of antibodies to tumor cells exposed to exofacial thiols; however, chemical modification is still required.

The direct targeting of extracellular redox states by antibodies is a significant challenge. Unlike conventional antigens, such as peptides or glycans, redox states resemble haptens with inherently lower structural diversity, which poses a challenge for antibody development by limiting the range of unique epitopes available for high-affinity and selective binding. There are very few examples of antibodies that directly target the redox states of cysteine, and most recognize oxidized forms. However, these antibodies demonstrate a high specificity towards distinct cysteine redox states. For example, several validated antibodies specifically bind to S-nitrosocysteine [125,126], sulfenic acid [127], and sulfinic or sulfonic acid [128], all of which are oxidation products of cysteine. The only example of an antibody that interacts with the reduced form of cysteine is a nanobody (Nb) that exposes a highly reactive cysteine in its complementarity-determining region 3 (CDR3) [65]. We could demonstrate how this Nb, denoted “CB2,” which has no identified target, relies on altered cell surface redox states to bind specifically to B cell non-Hodgkin lymphoma (BCL) and breast cancer cells. Notably, a fraction of Nb also undergoes thiol-mediated uptake via clathrin-dependent endocytosis, a mechanism that has been successfully exploited for drug delivery *in vitro* [65]. Interestingly, CB2 expression as a Chimeric Antigen Receptor (CAR) on T cells enables specific destruction of various BCL subtypes while sparing healthy cells based solely on their abnormal cell surface thiol levels [129]. The introduction of a similar single cysteine mutation to CDR3 in the clinically used anti-CD19 antibody fragment FMC63-CAR resulted in bispecific CAR-T cell activity. Surprisingly, ^Cys^CD19-CAR-T cells demonstrated cytotoxicity against both CD19-positive and CD19-negative BCL *in vitro*. In a mouse BCL model, ^Cys^CAR-T cells mitigated antigen escape by suppressing tumor growth and prolonging mouse survival without inducing systemic toxicity [129]. Moreover, as many cancer types exhibit an altered extracellular redox microenvironment, engineering similar cysteine motifs onto antibodies and antibody fragments could enable simultaneous specificity against their target antigen and altered exofacial thiols or disulfide levels in cancer cells, leading to the development of novel cancer theranostics.

### 3.6 Conclusions

Redox-targeted theranostics leverage a unifying principle: many cancers display altered extracellular redox states that can be exploited for selective delivery, retention, or activation of therapeutic and diagnostic agents. The examples discussed in the previous sections converge on a shared mechanism, yet they differ substantially in their chemical reactivity, biological behavior, and translational feasibility.

Small-molecule and inorganic scaffolds such as COCs, CPDs, selenite, and arsenic-based compounds benefit from rapid, efficient, and often endocytosis-independent cell entry [[95], [96], [97], [98],[100], [101], [102], [103], [104], [105], [106], [107]]. Their high redox reactivity enables traceless release of cargo or intracellular activation, but this same reactivity might pose challenges for *in vivo* use. Highly strained disulfides or inorganic redox drugs may undergo premature exchange or degradation in blood or off-target tissues, reducing effective tumor exposure. Inorganic compounds such as selenite or ATO already have partial clinical relevance, yet their systemic toxicity limits broad application [105,106,[130], [131], [132]]. On the other hand, no COC/CPD-based therapeutics have yet entered clinical trials.

Liposomes and nanoparticles offer improved stability and tunability, and their surfaces can be decorated with thiol-reactive groups (maleimide, PDS, COCs) to combine redox-dependent targeting with controlled pharmacological behavior [90,[108], [109], [110], [111], [112], [113], [114], [115], [116], [117], [118]]. Compared to small molecules, these systems permit modular functionalization (e.g., folate ligands, TLR agonists, fluorophores) and enable simultaneous targeting of exofacial thiols and tumor-associated receptors. However, nanoparticle systems must overcome barriers such as biodistribution, immune clearance, and manufacturing complexity [[133], [134], [135]]. While several redox-targeted nanoparticle systems show strong *in vivo* tumor retention and therapeutic benefit [109,[115], [116], [117]], none have advanced to clinical testing.

COC-functionalized peptides and biscysteine motifs allow efficient thiol-mediated uptake with good biocompatibility [119,120,122,123], yet their susceptibility to serum proteases and short serum half-life remain challenges for systemic therapy [136,137]. Antibodies and cysteine-engineered CARs illustrate the potential of combining redox-targeting with immunotherapy, as demonstrated by CB2 and the concept of cysteine-engineered CAR-T cells [65,129]. However, antibodies cannot be broadly applied to redox states because redox signatures present low epitope diversity, and the design of cysteine-engineered redox-sensing antibodies poses different challenges, most prominently the balance between original antigen specificity and redox reactivity [129]. Moreover, systemic administration of redox-reactive antibodies raises concerns about off-target disulfide exchange and unpredictable biodistribution, which remains to be examined *in vivo*.

Overall, redox-targeted theranostics form a coherent but diverse technological landscape unified by the exploitation of altered extracellular redox states in cancer. While preclinical progress is substantial, translation remains limited by challenges related to stability, systemic reactivity, and pharmacokinetics. Moreover, many studies reviewed here rely on very general cancer models, e.g. HeLa cells, and a broader applicability towards different cancer types as well as patient-derived specimens remains to be tested. Future work integrating controlled redox-reactive chemistries with clinically validated delivery platforms, improved *in vivo* characterization of tumor redox heterogeneity, and standardized comparative assessments will be essential to advance redox-targeted strategies toward clinical application.

## 4 Summary

**Fig. 3:**
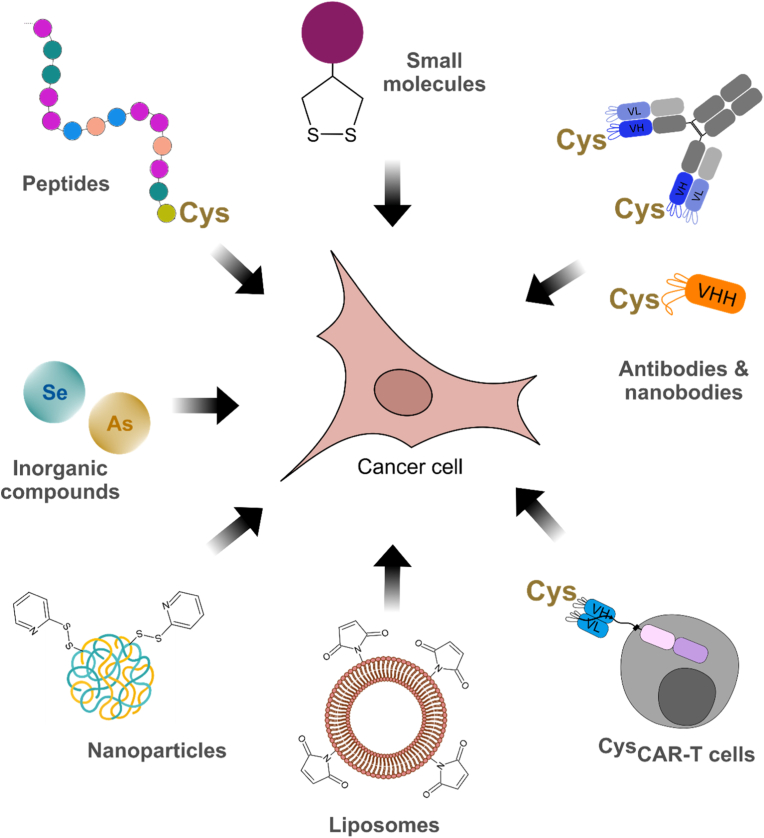
**Redox-directed theranostics of cancer cells**. A schematic overview about the different approaches to target cancer cells via altered extracellular redox states.

The altered cell surface redox environment of cancer provides compelling and underexploited opportunities for therapeutic and diagnostic targeting. One of the most promising strategies in this context is thiol targeting, which leverages elevated expression of exofacial thiol groups and redox-active enzymes in malignant cells. This approach has shown considerable potential across various molecular platforms, from small molecules and nanoparticles to peptides, proteins, and antibodies (Fig. 3). Thiol targeting offers several distinct advantages over conventional cancer therapy.

A key advantage of thiol targeting is its independence from specific antigens, which are often variable between cancer types, patient populations, or even within the same tumor over time [82,84]. Traditional immunotherapies depend on well-defined tumor-associated antigens (TAAs), which can be downregulated or lost through immune evasion mechanisms [83]. In contrast, thiol targeting exploits the biochemical signature of the tumor microenvironment, particularly its redox state, rather than discrete antigens. Elevated levels of cell-surface thiols are observed in a wide array of cancers, including breast cancer, B-CLL, prostate cancer, and fibrosarcoma. These thiol signatures are relatively conserved features of malignancy and correlate with invasiveness, therapy resistance, and metastatic potential. Thiol targeting offers a universal and robust mechanism for cancer recognition and therapy. Combining thiol-directed targeting of cancer cells with conventional immunotherapy has recently emerged as a promising research direction supported by encouraging preclinical results [65,129]. In this context, cysteine-engineered CAR designs demonstrate that the introduction of a single exposed cysteine into the CDR3 region enables CAR-T cells to engage altered extracellular redox states of B cell non-Hodgkin lymphoma in addition to their classical antigen specificity [129]. This redox-dependent interaction allows antigen-independent recognition of malignant cells and thereby addresses a major limitation of current CAR-T therapies, namely their susceptibility to antigen escape [138,139]. These results suggest that the integration of redox-sensitive targeting into established immunotherapeutic frameworks may provide a versatile strategy to enhance treatments against dynamically evolving tumors. However, reduced efficacy *in vivo* remains an important drawback, potentially caused by caused by cysteine oxidation or *cis*-interactions between reactive thiols [129]. Future improvements may include more robust redox-reactive motifs such as cysteine-glycine-cysteine (CGC) [123], optimizing the redox environment during CAR-T cell preparation and infusion, or refining cysteine placement through structure-guided engineering to enhance stability and activity.

Another significant benefit of thiol-targeted strategies is their enhanced tissue penetration capacity. This is particularly relevant for the treatment of solid tumors, which are notoriously difficult to access because of their dense extracellular matrices and high interstitial pressures. Several thiol-reactive systems have demonstrated superior intratumoral distribution compared with conventional approaches [100,115,118,121]. These findings suggest that thiol-targeted delivery systems not only improve cellular uptake but also overcome physiological barriers to reach deeper tumor regions.

A particularly important insight from recent studies is the minimal structural requirements for thiol-directed targeting and thiol-mediated uptake. The incorporation of a single cysteine residue into peptides or proteins has been shown to significantly improve cellular uptake in a thiol-dependent manner [65,122]. However, biscysteine modifications offer a greater efficiency. For example, the insertion of a CGC motif, which promotes intramolecular disulfide cycling, further enhances thiol-mediated entry [123]. Such motifs likely facilitate more favorable interaction kinetics with cell-surface thiols and disulfides, thereby promoting faster and more efficient translocation.

The exact mechanism by which thiol-targeted entities enter cells varies, depending on the structure and reactivity of the delivery vehicle. Thiol-mediated uptake can occur via multiple routes including direct translocation and endocytosis. For example, some COCs such as ETP and BPS undergo direct cytosolic entry, bypassing classical endocytic pathways through dynamic covalent exchange with exofacial thiols [96,97]. In contrast, certain thiolated lipid nanoparticles and cysteine-modified proteins undergo clathrin-mediated endocytosis for entry. For example, a nanobody with a reactive cysteine was shown to bind exofacial thiols and enter cells via clathrin-dependent endocytosis [65]. Thus, thiol-mediated uptake encompasses a spectrum of uptake routes, suggesting that endocytosis is not a strict requirement, but a viable mechanism.

The molecular basis of thiol-mediated uptake at the cell surface involves dynamic covalent interactions, primarily between the thiol and disulfide groups. Two main mechanisms underpin this process: thiol–disulfide exchange and thiol–thiol interactions. The former is well documented and constitutes the core of many redox-responsive systems. In thiol–disulfide exchange, an incoming thiolate nucleophile attacks a disulfide bond on the target surface or carrier, leading to bond reshuffling and molecular translocation [86]. Although less common, thiol–thiol interactions may also contribute to uptake, or at least initial surface binding [85].

Besides these advantages, redox-targeted therapies have not yet progressed to clinical application. Existing platforms, including COCs, CPDs, thiol-reactive liposomes, nanoparticles, and cysteine-engineered antibodies, demonstrate promising mechanistic activity *in vitro* and in animal models, but their translational potential is hindered by challenges such as systemic stability [140], off-target reactivity [141,142], and *in vivo* tumor redox heterogeneity [143]. To enable clinical advancement, future strategies will need to incorporate more controlled and biocompatible redox-reactive chemistries, integrate redox targeting with clinically validated delivery systems, and systematically address pharmacokinetics, tumor selectivity, and redox-state variability across patients. The incorporation of redox-reactive therapeutics into immunotherapy, as discussed above, may provide an effective strategy for targeted delivery to the tumor site while reducing the risk of premature or off-target reactivity.

Collectively, the evidence presented in this review supports a paradigm in which redox targeting represents a versatile and powerful strategy for cancer theranostics. By exploiting the elevated levels of surface thiols and redox-active enzymes, novel anticancer tools can bypass the limitations of antigen-specific targeting. Redox-mediated systems offer advantages, such as improved tissue penetration, broad applicability across tumor types, and the ability to facilitate advanced delivery systems, including vaccines, liposomes, and nanoparticles. Moreover, the modularity of thiol-mediated uptake allows fine-tuning of delivery vehicles based on cysteine content, disulfide configuration, and scaffold rigidity. Whether uptake occurs via direct translocation or endocytosis, the shared initiating event is an interaction with cell surface redox states that might also allow to distinguish malignant and normal cells. Integrating thiol-targeting strategies with other modalities, such as immunotherapy, could further enhance efficacy and selectivity. Ultimately, the redox landscape of tumors provides a robust and biochemically distinct feature that can be systematically harnessed to improve the precision and potency of cancer treatment. Despite their conceptual advantages, redox-targeted therapies remain at the preclinical stage and continue to face translational barriers, including premature reactivity and heterogeneous tumor redox states. However, emerging advances in molecular design and targeted delivery offer a steadily improving foundation for addressing these limitations and may, in time, support the progression of redox-guided therapeutics toward clinical application.

## Funding

The financial support from the Max Planck Society is gratefully acknowledged. This work was further supported by Deutsche Forschungsgemeinschaft (RTG2046 for J.L.).

## CRediT authorship contribution statement

**Jost Lühle:** Conceptualization, Writing – original draft. **Peter H. Seeberger:** Funding acquisition, Project administration, Supervision. **Oren Moscovitz:** Conceptualization, Supervision, Writing – review & editing.

## Declaration of competing interest

The authors declare the following competing financial interest(s): JL, PHS, and OM have patents pending on cysteine-engineered antigen-binding modules and the method of cysteine-engineered CAR-T cells.

## Data Availability

No data was used for the research described in the article.
